# Bioactive Potential of *Nepenthes miranda* Flower Extracts: Antidiabetic, Anti-Skin Aging, Cytotoxic, and Dihydroorotase-Inhibitory Activities

**DOI:** 10.3390/plants14162579

**Published:** 2025-08-19

**Authors:** Kuan-Ming Lai, Yen-Hua Huang, Yi Lien, Cheng-Yang Huang

**Affiliations:** 1 Hemato Oncology Division Department of Internal Medicine, Changhua Christian Hospital, Changhua 500, Taiwan; 2 Department of Biomedical Sciences, Chung Shan Medical University, Taichung City 402, Taiwan; 3 Department of Biological Sciences, Purdue University, West Lafayette, IN 47907, USA; 4 Department of Medical Research, Chung Shan Medical University Hospital, Taichung City 402, Taiwan

**Keywords:** *Nepenthes miranda*, dihydroorotase, antioxidant, antibacterial, antidiabetic, anticancer, A431, elastase, hyaluronidase, tyrosinase

## Abstract

Carnivorous plants have garnered attention as sources of pharmacologically active compounds, yet their floral tissues remain largely underexplored. In this study, we investigated the bioactive properties of *Nepenthes miranda* flower extracts prepared using water, methanol, ethanol, and acetone. Among these, the ethanol extract exhibited the highest total phenolic content (18.2 mg GAE/g), flavonoid content (68.9 mg QUE/g), and antioxidant activity (DPPH IC_50_ = 66.9 μg/mL), along with strong antibacterial effects against *Escherichia coli* and *Staphylococcus aureus*. Cosmetically relevant enzyme inhibition assays revealed significant activity against tyrosinase (IC_50_ = 48.58 μg/mL), elastase (IC_50_ = 1.77 μg/mL), and hyaluronidase (IC_50_ = 7.33 μg/mL), supporting its potential as an anti-skin aging agent. For antidiabetic evaluation, the ethanol extract demonstrated potent α-glucosidase inhibition (IC_50_ = 24.53 μg/mL), outperforming standard inhibitors such as acarbose and quercetin. The extract also displayed marked cytotoxicity against A431 epidermoid carcinoma cells (IC_50_ = 90.61 μg/mL), inducing dose-dependent apoptosis, inhibiting cell migration and colony formation, and causing significant DNA damage as shown by comet assay. Furthermore, the ethanol extract strongly inhibited the activity of purified human dihydroorotase (IC_50_ = 25.11 μg/mL), indicating that disruption of pyrimidine biosynthesis may underlie its anticancer activity. Overall, this study provides the first characterization of *N. miranda* flower extracts, particularly the ethanol fraction, as a promising source of multifunctional bioactive compounds with possible applications in cosmetics, antidiabetic therapy, and cancer treatment.

## 1. Introduction

Carnivorous plants have long fascinated scientists due to their unique adaptations to nutrient-poor environments, from which they derive essential nutrients by capturing and digesting prey [1,2]. Among them, species of the genus *Nepenthes* exhibit specialized pitcher-shaped leaves that function as passive pitfall traps [3,4,5]. These plants have attracted attention not only for their ecological and evolutionary significance but also for their ethnobotanical uses [3]. For example, the boiled roots of *N. ampullaria* and *N. gracilis* are traditionally used by Malaysian tribes to treat stomach aches [3]. In recent years, increasing scientific interest has focused on the pharmacological properties of *Nepenthes* species, with bioactive compounds identified in extracts from pitchers, stems, and leaves, including those with notable anticancer activity [6,7,8,9,10,11]. However, despite the morphological and biochemical diversity of *Nepenthes*, the pharmacological potential of their floral tissues remains largely unexplored and warrants investigation. *Nepenthes miranda*, a horticulturally derived hybrid of *N. maxima* and *N. northiana*, is known for its distinct physiological characteristics and has demonstrated various biological activities [12,13]. Extracts from its stem, leaves, and pitchers have shown antibacterial, antioxidant, and cytotoxic effects against a range of cancer cell lines, including mouse B16F10 melanoma, 4T1 breast cancer cells, and human lung (PC9, H838, H1975, A549) and oral (Ca9-22, CAL27) cancer cells, with the stem extract generally exhibiting the highest activity [6,10,14,15,16,17]. However, harvesting the stem extract results in the destruction of the plant, posing sustainability concerns for its long-term use. Since floral extracts from various plant species, particularly those rich in phenolic and flavonoid compounds, are increasingly recognized for their therapeutic and cosmeceutical value [18,19,20,21,22], the flower extracts of *Nepenthes* species represent a promising yet underexplored source of bioactive compounds that merit further pharmacological investigation.

Tissues of *Nepenthes* species are known to be rich sources of secondary metabolites with potential pharmaceutical and medical applications [7,8]. Compounds identified from various *Nepenthes* extracts include phenolic acids and their derivatives such as gallic, protocatechuic, chlorogenic, ferulic, p-coumaric, hydroxybenzoic, vanillic, syringic, and caffeic acids, as well as vanillin; flavonoids such as myricetin, quercetin, and kaempferol derivatives; anthocyanins including delphinidin-3-O-glucoside, cyanidin-3-O-glucoside, and cyanidin; naphthoquinones such as plumbagin, droserone, and 5-O-methyl droserone; and various volatile organic compounds. The presence of these unique flavonoids and phenolic compounds is commonly associated with antioxidant, antimicrobial, anti-aging, and cytotoxic activities. However, the chemical composition of flowers, particularly their volatile scent molecules that serve to attract insect prey, remains poorly characterized. Therefore, in this study, the flower extract of *N. miranda* was analyzed using GC–MS to obtain a preliminary understanding of its components.

Diabetes mellitus is a chronic metabolic disorder characterized by impaired glucose metabolism and persistent hyperglycemia [23,24,25]. Type 2 diabetes mellitus (T2DM), which accounts for approximately 90–95% of all diabetes cases, is primarily caused by insulin resistance and inadequate insulin secretion. One therapeutic strategy for managing T2DM involves delaying glucose absorption by inhibiting carbohydrate-hydrolyzing enzymes, particularly α-glucosidase, which plays a crucial role in the final step of carbohydrate digestion by converting oligosaccharides into glucose in the small intestine [26]. Inhibition of α-glucosidase reduces postprandial blood glucose spikes and improves glycemic control, making it a valuable target for antidiabetic drug development. Accordingly, identifying additional inhibitors, including functional plant extracts, against α-glucosidase may contribute to improved treatment strategies for T2DM.

Skin aging is a multifaceted process driven by both intrinsic aging and external factors, resulting in common signs such as wrinkles, reduced elasticity, and dryness [27,28,29,30,31]. Natural compounds have garnered attention for their potential to counteract these effects by inhibiting enzymes that degrade key components of the skin matrix [32]. Among these, tyrosinase [33,34,35,36], elastase [37,38], and hyaluronidase [38,39] play major roles in melanin production, elastin breakdown, and hyaluronic acid degradation, respectively. Their heightened activity is associated with pigmentation disorders, loss of firmness, and diminished skin hydration [40]. Moreover, several molecular pathways involved in aging have been implicated in the progression of cancer [41]. In this study, we explored the anti-skin aging potential of flower extracts from *N. miranda*, focusing on the ethanol extract, which demonstrated promising inhibitory effects on these enzymes. These findings suggest that the extract may help preserve skin tone, elasticity, and moisture, supporting its application as a functional ingredient in cosmetic formulations aimed at delaying or reducing skin aging.

Cancer remains a growing threat to global public health [42,43,44,45,46,47,48], with skin cancer representing a particularly prevalent and heterogeneous group of malignancies [49,50,51]. Approximately one in every three diagnosed cancers worldwide is a form of skin cancer [52]. Skin cancers are generally classified into two major categories: melanoma skin cancer (MSC) and non-melanoma skin cancers (NMSCs), with the latter being the most common. NMSCs primarily include basal cell carcinoma (BCC) and squamous cell carcinoma (SCC), both of which originate from epidermal keratinocytes. These represent the most frequently diagnosed malignancies in humans, particularly among Caucasian populations, and their incidence continues to rise globally [53,54]. Given the long-standing use of plant-derived compounds in traditional medicine and their growing relevance in modern therapeutics [55,56,57,58], with approximately 60% of current anticancer drugs derived from or inspired by natural products [59,60], we investigated the potential cytotoxic effects of *N. miranda* flower extracts on A431 cells. A431 cells, derived from a human epidermoid carcinoma (a type of SCC), are widely used as a model for skin cancer and epidermal growth factor receptor (EGFR)-targeted studies due to their high EGFR expression [61]. In this study, we evaluated whether the flower extracts could inhibit A431 cell survival, migration, and proliferation and induce apoptosis and DNA damage. Based on the cytotoxicity observed, the flower extracts of *N. miranda* may offer promising anticancer potential for future therapeutic applications.

To identify potential molecular targets underlying the anticancer effects of *N. miranda* flower extracts, we investigated whether dihydroorotase could be one such target. Dihydroorotase is a key enzyme in the de novo pyrimidine biosynthesis pathway [62,63] and, along with dihydropyrimidinase [64,65,66], allantoinase [67,68,69], and hydantoinase [70,71,72], belongs to the cyclic amidohydrolase family [73,74,75,76]. In humans, dihydroorotase activity resides within the multifunctional enzyme CAD, which also exhibits carbamoyl phosphate synthetase and aspartate transcarbamoylase activities [77,78]. CAD is frequently upregulated in cancer cells due to their elevated demand for nucleotide synthesis required for rapid proliferation [79,80,81]. Interestingly, a recent study has reported that afatinib [82], a clinically approved EGFR inhibitor used in lung cancer treatment, is also capable of inhibiting CAD enzymatic activity [83]. Given that A431 cells overexpress EGFR and were sensitive to the *N. miranda* flower extracts, we hypothesized that dihydroorotase might serve as a relevant target. Since normal cells typically exhibit lower nucleotide synthesis rates than cancer cells, inhibition of dihydroorotase could provide selective anticancer effects. Our results showed that the ethanol extract of *N. miranda* significantly suppressed the enzymatic activity of human dihydroorotase, suggesting that interference with pyrimidine biosynthesis may be one mechanism underlying its cytotoxicity. Overall, *N. miranda* flower extracts may represent a promising source of multifunctional bioactive compounds with potential applications in cosmetics, diabetes management, and cancer therapy.

## 2. Results

### 2.1. Total Phenolic Content (TPC)

**Figure 1:**
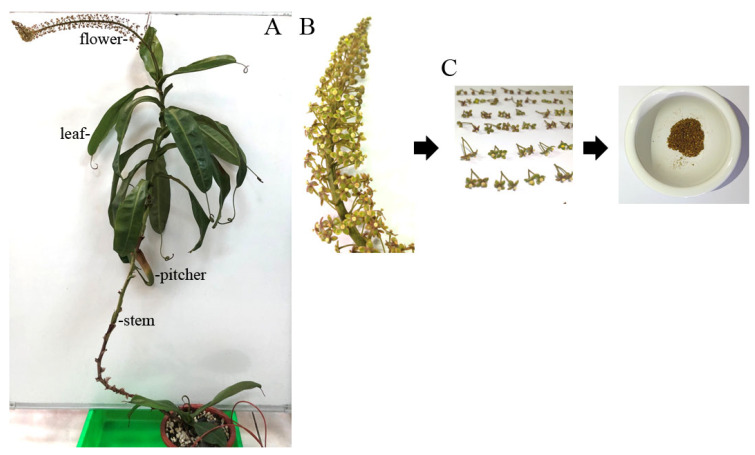
Preparation of different extracts from *N. miranda*. (**A**) Whole plant of *N. miranda*, showing its distinct morphological structures including leaves, stem, pitcher, and flower. To fully display the plant structure, the stem was gently suspended using a small hook. (**B**) A close-up view of the flower cluster at full bloom. (**C**) Harvested flowers including pedicels were dried and ground into powder for subsequent extraction and pharmacological analysis.

The pharmacological potential of the *N. miranda* flower remains largely unexplored, particularly regarding its antioxidant, cytotoxic, anti-aging, and antidiabetic properties. To address this knowledge gap, flower extracts of *N. miranda* were prepared to evaluate their bioactivity. The plant, characterized by distinct morphological structures including leaves, stems, pitchers, and flowers, was used for extraction with water, methanol, ethanol, and acetone (Figure 1A). Flower clusters were harvested at full bloom (Figure 1B), and the flowers, including pedicels, were collected, dried, and pulverized into powder for subsequent extraction and pharmacological analyses (Figure 1C). Considering that many polyphenols identified through in vitro and in vivo studies serve as potential drug candidates, the total phenolic content (TPC) of *N. miranda* flower extracts was analyzed (Table 1). TPC was quantified using the modified Folin–Ciocalteu method [84] and expressed as mg gallic acid equivalents per gram of dry extract (mg GAE/g). Among the flower extracts, TPC ranged from 8.6 mg GAE/g for the water extract to 18.2 mg GAE/g for the ethanol extract, the latter being the highest. For comparison, other plant parts were also extracted and analyzed. The highest TPC among these was found in the stem acetone extract at 16.2 mg GAE/g, which was lower than the TPC values of both the ethanol and methanol extracts of the flower.

### 2.2. Total Flavonoid Content (TFC)

Flavonoids are natural products known for their structure-dependent biological and pharmacological activities. Accordingly, we determined the total flavonoid content (TFC) of the flower extract of *N. miranda* (Table 2). TFC was quantified using the aluminum chloride colorimetric method [85]. The TFC values for flower extracts ranged from 10.2 mg QUE/g for the water extract to 68.9 mg QUE/g for the ethanol extract. Notably, the ethanol extract exhibited the highest TFC among all tested extracts, including those from other plant parts.

### 2.3. Antioxidant Activity of the Flower Extracts

Antioxidant activity is often linked to the presence of bioactive phytochemicals, particularly phenolics and flavonoids. Given the high levels of these compounds in the flower extracts of *N. miranda*, especially in the ethanol extract, we proceeded to assess their antioxidant potential (Table 3). The evaluation was conducted using the 1,1-diphenyl-2-picrylhydrazyl (DPPH) radical scavenging assay, a widely accepted method for measuring free radical inhibition. The antioxidant capacities were determined by calculating the IC_50_ values from DPPH scavenging curves. The IC_50_ for the water extract could not be reliably determined due to insufficient activity. Among the tested flower extracts, the ethanol extract demonstrated the strongest antioxidant potential, with an IC_50_ value of 66.9 μg/mL.

### 2.4. Antibacterial Activity of the Flower Extracts

The antibacterial properties of various flower extracts were assessed using the agar well diffusion assay, with inhibition zones used as a measure of activity (Table 4). *Escherichia coli* and *Staphylococcus aureus* were selected as representative Gram-negative and Gram-positive bacteria, respectively. The extracts (10 mg; 100 μL of 100 mg/mL) displayed variable antibacterial effects, with inhibition zones ranging from 0 to 29 mm. The water extract showed no detectable antibacterial activity against either strain. The acetone extract exhibited only minimal inhibition. In contrast, the ethanol extract demonstrated the strongest antibacterial effects, producing inhibition zones of 29 mm for *E. coli* and 27 mm for *S. aureus*, suggesting comparable efficacy against both bacterial types.

### 2.5. Tyrosinase Inhibitory Activity of the Flower Extracts

**Figure 2:**
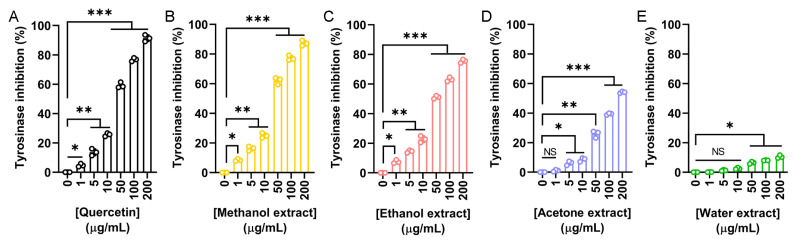
Tyrosinase inhibitory activity of the flower extracts. Tyrosinase inhibition was assessed using the dopachrome assay with L-DOPA as the substrate. (**A**) Quercetin was used as a positive control. Flower extracts were prepared using (**B**) methanol, (**C**) ethanol, (**D**) acetone, and (**E**) water. Each extract was tested at increasing concentrations (0, 1, 5, 10, 50, 100, and 200 μg/mL). The methanol extract exhibited the highest tyrosinase inhibition, even surpassing that of quercetin at some concentrations. Data represent mean ± SD (*n* = 3). Levels of statistical significance are indicated by * *p* < 0.05, ** *p* < 0.01, and *** *p* < 0.001 in comparison to the control group; NS, not significant.

To explore the potential cosmetic applications of *N. miranda* flower extracts, their ability to inhibit tyrosinase was evaluated using the modified dopachrome method. Tyrosinase is a key enzyme in melanin biosynthesis. Since melanin overproduction is associated with skin aging and hyperpigmentation, targeting tyrosinase is a common strategy in skin-whitening and anti-aging formulations. Quercetin, a known natural tyrosinase inhibitor, was used as a positive control (Figure 2A). The flower extracts demonstrated varying levels of tyrosinase inhibition depending on the solvent used: methanol (Figure 2B), ethanol (Figure 2C), and acetone (Figure 2D) showed dose-dependent inhibition, while the water extract (Figure 2E) exhibited minimal activity. The IC_50_ values were calculated as 39.07 μg/mL for quercetin, 37.04 μg/mL for the methanol extract, 48.58 μg/mL for the ethanol extract, and 172.43 μg/mL for the acetone extract (Table 5). The IC_50_ for the water extract could not be determined due to its negligible inhibition. Accordingly, the methanol extract demonstrated the strongest inhibitory effect, even surpassing quercetin. Thus, the flower extract of *N. miranda*, particularly the methanol or ethanol fraction, may have potential for cosmetic applications in managing skin pigmentation and promoting skin lightening.

### 2.6. Elastase Inhibitory Activity of the Flower Extracts

**Figure 3:**
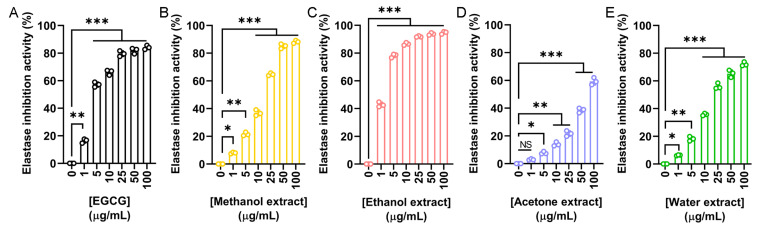
Elastase inhibitory activity of the flower extracts. Elastase activity assay was assessed using N-Succinyl-Ala-Ala-Ala-p-nitroanilide as the substrate. (**A**) EGCG was used as a positive control. Flower extracts were prepared using (**B**) methanol, (**C**) ethanol, (**D**) acetone, and (**E**) water. Each extract was tested at increasing concentrations (0, 1, 5, 10, 25, 50, and 100 μg/mL). The ethanol extract exhibited the strongest elastase inhibition, exceeding even the potency of EGCG. Data represent mean ± SD (*n* = 3). Levels of statistical significance are indicated by * *p* < 0.05, ** *p* < 0.01, and *** *p* < 0.001 in comparison to the control group; NS, not significant.

The elastase inhibitory potential of *N. miranda* flower extracts was evaluated to assess their relevance in skin aging prevention. Elastase is a serine protease that degrades elastin and other extracellular matrix proteins. Overactivity of elastase contributes to reduced skin elasticity, hydration loss, and structural damage to epithelial tissues. Using epigallocatechin gallate (EGCG) as a positive control (Figure 3A), we investigated the inhibitory activity of flower extracts prepared with methanol (Figure 3B), ethanol (Figure 3C), acetone (Figure 3D), and water (Figure 3E). The IC_50_ values obtained were 4.25 μg/mL for EGCG, 17.06 μg/mL for the methanol extract, 1.77 μg/mL for the ethanol extract, 77.94 μg/mL for the acetone extract, and 20.59 μg/mL for the water extract (Table 5). Accordingly, the ethanol extract exhibited the strongest elastase inhibition, exceeding even the potency of EGCG. Thus, the ethanol extract may have promising anti-skin aging applications by preserving elastin integrity and preventing wrinkle formation.

### 2.7. Hyaluronidase Inhibitory Activity of the Flower Extracts

**Figure 4:**
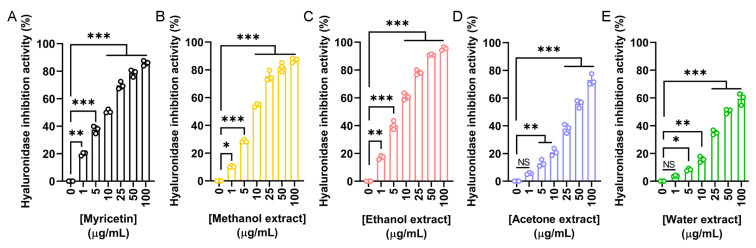
Hyaluronidase inhibitory activity of the flower extracts. Hyaluronidase activity assay was assessed using hyaluronic acid as the substrate. (**A**) Myricetin was used as a positive control. Flower extracts were prepared using (**B**) methanol, (**C**) ethanol, (**D**) acetone, and (**E**) water. Each extract was tested at increasing concentrations (0, 1, 5, 10, 25, 50, and 100 μg/mL). The ethanol extract exhibited the strongest hyaluronidase inhibition. Data represent mean ± SD (*n* = 3). Levels of statistical significance are indicated by * *p* < 0.05, ** *p* < 0.01, and *** *p* < 0.001 in comparison to the control group; NS, not significant.

To evaluate the anti-aging and dermal protection potential of *N. miranda* flower extracts, their ability to inhibit hyaluronidase was assessed. Hyaluronidase is an enzyme responsible for the degradation of hyaluronic acid, a key component of the extracellular matrix that maintains skin hydration and elasticity. Excessive hyaluronidase activity is associated with skin aging and inflammation. Myricetin was used as a positive control (Figure 4A), showing a concentration-dependent inhibition with an IC_50_ of 9.74 μg/mL. The IC_50_ values obtained were 8.58 μg/mL for the methanol extract (Figure 4B), 7.33 μg/mL for the ethanol extract (Figure 4C), 41.97 μg/mL for the acetone extract (Figure 4D), and 49.28 μg/mL for the water extract (Figure 4E). Accordingly, the ethanol extract exhibited the strongest hyaluronidase inhibition among the flower extracts (Table 5). The methanol extract also demonstrated potent activity. Such hyaluronidase inhibition may help preserve skin moisture and structure, supporting the potential application of these extracts in cosmetic formulations aimed at preventing skin aging.

### 2.8. Antidiabetic Potential of N. miranda Flower Extracts via α-Glucosidase Inhibitory Activity

**Figure 5:**
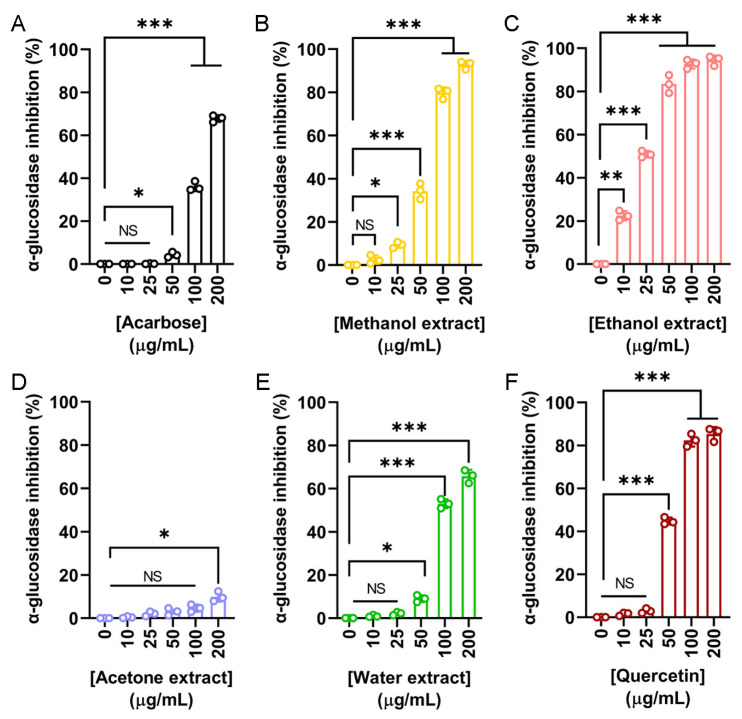
α-Glucosidase inhibitory activity of the flower extracts. α-Glucosidase activity assay was assessed using *p*-nitrophenyl-α-D-glucopyranoside as the substrate. (**A**) Acarbose was used as a positive control. Flower extracts were prepared using (**B**) methanol, (**C**) ethanol, (**D**) acetone, and (**E**) water. Each extract was tested at increasing concentrations (0, 10, 25, 50, 100, and 200 μg/mL). (**F**) Quercetin was used as a second, more potent standard inhibitor, for comparison. The ethanol extract exhibited the strongest α-glucosidase inhibition. Data represent mean ± SD (*n* = 3). Levels of statistical significance are indicated by * *p* < 0.05, ** *p* < 0.01, and *** *p* < 0.001 in comparison to the control group; NS, not significant.

In addition to cosmetic applications, the potential antidiabetic properties of *N. miranda* flower extracts were also investigated. α-Glucosidase is a key enzyme involved in the hydrolysis of oligosaccharides, contributing to postprandial hyperglycemia. Inhibitors of α-glucosidase are clinically used to delay glucose absorption and manage type 2 diabetes mellitus (T2DM). To assess this activity, the inhibitory effects of flower extracts were compared to two known α-glucosidase inhibitors, acarbose and quercetin. Acarbose served as the primary positive control (Figure 5A), showing a dose-dependent inhibition with an IC_50_ of 143.71 μg/mL. The methanol (Figure 5B), ethanol (Figure 5C), acetone (Figure 5D), and water (Figure 5E) extracts were tested at various concentrations. The ethanol extract exhibited the most potent α-glucosidase inhibition, with an IC_50_ of 24.53 μg/mL, significantly lower than that of acarbose. The methanol and water extracts also demonstrated inhibitory effects, with IC_50_ values of 67.18 and 96.69 μg/mL, respectively. In contrast, the acetone extract showed minimal activity, and its IC_50_ could not be determined (Table 5). Given the strong α-glucosidase inhibitory activity observed in three of the flower extracts, which surpassed that of the positive control acarbose, a second, more potent standard inhibitor, quercetin, was also included for comparison (Figure 5F). Quercetin demonstrated superior inhibition compared to acarbose, with an IC_50_ of 56.85 μg/mL. However, this value remained higher than that of the ethanol extract, indicating that the ethanol extract possesses even greater inhibitory potential. Accordingly, these results demonstrate the strong in vitro α-glucosidase inhibitory activity of *N. miranda* flower extracts, particularly the ethanol fraction, suggesting its potential for further investigation as a candidate for glucose control or T2DM management.

### 2.9. Anticancer Potential

**Figure 6:**
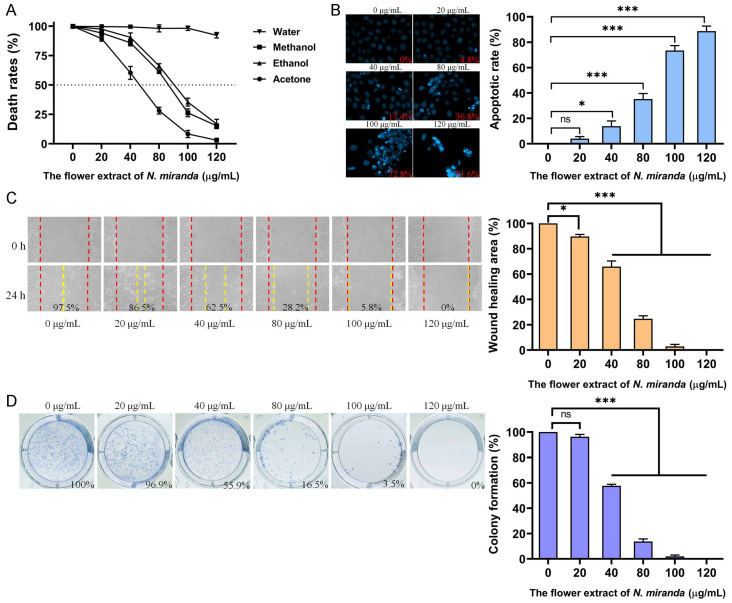
Evaluation of the anticancer potential of *N. miranda* flower extracts in A431 human epidermoid carcinoma cells. (**A**) Cytotoxicity of flower extracts (0–120 μg/mL) from water, methanol, ethanol, and acetone was assessed using the MTT assay. The methanol, ethanol, and acetone extracts significantly reduced cell viability in a dose-dependent manner, while the water extract showed minimal effect. (**B**) Apoptosis induction was evaluated using Hoechst 33342 staining. Representative fluorescence images and quantified apoptotic rates revealed a dose-dependent increase in chromatin condensation and nuclear fragmentation upon treatment with the ethanol extract. (**C**) The wound-healing assay was used to assess the inhibitory effect of the ethanol extract on cell migration. Quantitative analysis of wound closure demonstrated significant dose-dependent suppression of A431 cell motility after 24 h. (**D**) Proliferative capacity was analyzed using a clonogenic formation assay. Ethanol extract treatment led to a marked, dose-dependent reduction in colony formation, indicating impaired long-term proliferative potential of A431 cells. Data are presented as mean ± SD (*n* = 3). Levels of statistical significance are indicated by * *p* < 0.05 and *** *p* < 0.001 in comparison to the control group; ns, not significant.

Skin cancers, such as squamous cell carcinoma (SCC), are among the most aggressive malignancies. To evaluate the potential anticancer activity of *N. miranda* flower extracts, we examined their effects on the A431 cell line, a widely used human epidermoid carcinoma model for SCC (Figure 6). Cytotoxicity was first assessed using the MTT assay, which quantifies mitochondrial activity as an indicator of cell viability. As shown in Figure 6A, the methanol, ethanol, and acetone flower extracts significantly reduced A431 cell viability in a dose-dependent manner, with IC_50_ values of 86.49 ± 0.25 μg/mL, 90.61 ± 1.12 μg/mL, and 52.25 ± 5.11 μg/mL, respectively. The water extract did not exhibit notable cytotoxicity. Based on its strong performance in antioxidant activity (Table 3), antibacterial efficacy (Table 4), and enzyme inhibition (Table 5), the ethanol extract was selected for further investigation of its effects on apoptosis, migration, and proliferation. Apoptotic induction was evaluated using a chromatin condensation assay with Hoechst 33342 staining, a method that detects DNA fragmentation and condensation as indicators of programmed cell death. Ethanol extract treatment at concentrations of 0, 20, 40, 80, 100, and 120 μg/mL resulted in apoptotic rates of 0%, 4.8%, 17.4%, 36.8%, 72.8%, and 91.6%, respectively (Figure 6B), indicating a dose-dependent increase in apoptosis. To assess anti-migratory potential, we performed a wound-healing assay. Ethanol extract treatment significantly impaired A431 cell migration, with wound closure rates declining from 97.5% in the control group to 86.5%, 62.5%, 28.2%, 5.8%, and 0% following treatment with 20, 40, 80, 100, and 120 μg/mL, respectively (Figure 6C). Furthermore, clonogenic assay results demonstrated a strong dose-dependent inhibition of colony formation (Figure 6D). At concentrations of 20, 40, 80, 100, and 120 μg/mL, colony formation rates were reduced to 96.9%, 55.9%, 16.5%, 3.5%, and 0%, respectively, compared to 100% in the untreated control. While these results demonstrate significant in vitro cytotoxicity toward skin cancer cells, they are considered preliminary, as the effect on normal skin control cells was not evaluated in this study.

### 2.10. Genotoxic Effects of N. miranda Flower Extract on A431 Cells

**Figure 7:**
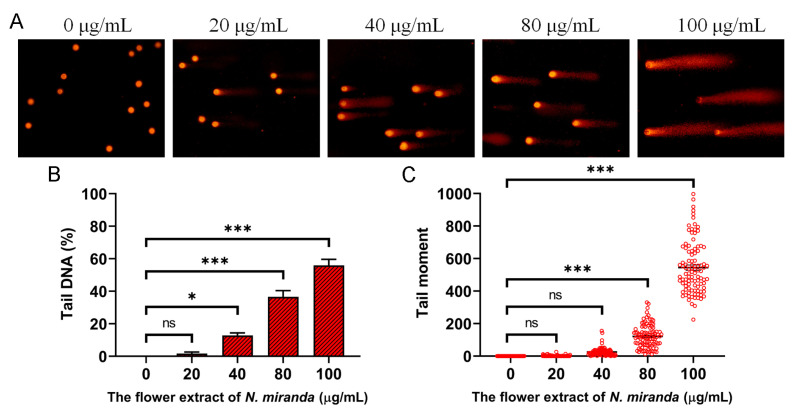
DNA damage induced by the ethanol flower extract of *N. miranda* in A431 cells. (**A**) Representative images of comet assay showing DNA fragmentation in A431 cells treated with different concentrations (0, 20, 40, 80, and 100 μg/mL) of the ethanol extract. Increased comet tail formation indicates enhanced DNA strand breaks. (**B**) Quantification of tail DNA (%) revealed a dose-dependent increase in DNA damage. (**C**) Tail moment analysis confirmed the concentration-dependent genotoxic effect of the extract. Data are presented as mean ± SD (*n* ≥ 100 comets per group). Levels of statistical significance are indicated by * *p* < 0.05 and *** *p* < 0.001 in comparison to the control group; ns, not significant.

Our investigation demonstrated that the ethanol extract of *N. miranda* flowers induces genotoxic stress in A431 cells, as assessed by single-cell gel electrophoresis (comet assay), a highly sensitive method for detecting DNA strand breaks at the single-cell level. A concentration-dependent increase in DNA damage was observed following treatment with the extract. During the assay, cells embedded in agarose underwent lysis, and subsequent electrophoresis allowed fragmented DNA to migrate, forming a comet tail as a hallmark of DNA damage. Cells treated with increasing concentrations of the extract exhibited longer and more intense comet tails compared to untreated controls, indicating elevated levels of DNA fragmentation (Figure 7A). The visual analysis revealed varying degrees of damage, ranging from negligible in the control group to extensive fragmentation in treated cells. Quantitatively, treatment with 0, 20, 40, 80, and 100 μg/mL of the extract resulted in tail DNA percentages of 0%, 1.7%, 12.9%, 36.6%, and 55.9%, respectively (Figure 7B), alongside a corresponding dose-dependent increase in tail moment values (Figure 7C). These findings suggest that the extract induces significant DNA damage, likely contributing to apoptosis and impaired cell viability. Accordingly, the genotoxic potential of the ethanol extract may underlie its anticancer activity, particularly through the induction of DNA-damage-mediated cell death in skin carcinoma cells.

### 2.11. Dihydroorotase Inhibitory Activity of the Flower Extracts

**Figure 8:**
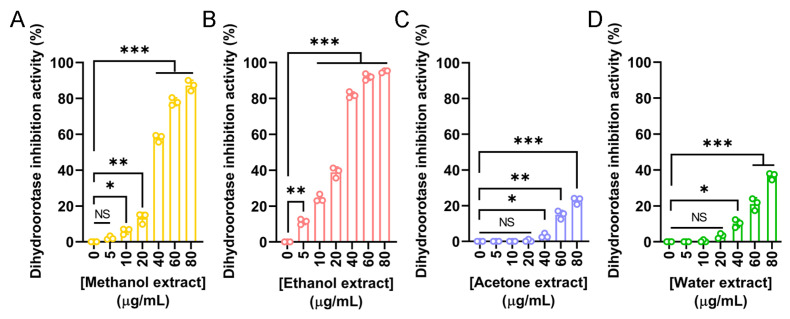
Inhibitory activity of *N. miranda* flower extracts against human dihydroorotase. Dihydroorotase inhibition was assessed using extracts prepared from *N. miranda* flowers with (**A**) methanol, (**B**) ethanol, (**C**) acetone, and (**D**) water across a concentration range of 0–80 μg/mL. The inhibitory activity was quantified based on the reduction in enzyme activity relative to untreated controls. Both methanol and ethanol extracts exhibited significant, dose-dependent inhibition of dihydroorotase activity, with the ethanol extract showing the strongest effect. Acetone and water extracts exhibited only mild or negligible inhibition. Data are presented as mean ± SD (*n* = 3). Levels of statistical significance are indicated by * *p* < 0.05, ** *p* < 0.01, and *** *p* < 0.001 in comparison to the control group; NS, not significant.

Dihydroorotase has recently emerged as a promising target for therapeutic intervention due to its central role in the de novo pyrimidine biosynthesis pathway, which is essential for the survival and proliferation of rapidly dividing cells, including cancer cells. Given the observed anticancer potential of the *N. miranda* flower extracts, we evaluated their ability to inhibit human dihydroorotase activity. Human dihydroorotase was expressed and purified using Ni-NTA affinity chromatography, as previously described [86]. Extracts prepared with methanol (Figure 8A), ethanol (Figure 8B), acetone (Figure 8C), and water (Figure 8D) were tested across a range of concentrations. Among these, the ethanol extract exhibited the strongest inhibitory effect, with an IC_50_ of 25.11 ± 0.77 μg/mL. The methanol extract also demonstrated substantial activity, with an IC_50_ of 36.48 ± 0.65 μg/mL. In contrast, the acetone and water extracts showed minimal inhibition, and their IC_50_ values could not be determined. These findings suggest that the anticancer activity of the flower extracts, particularly those prepared with ethanol and methanol, may be partially attributed to their inhibition of dihydroorotase and interference with pyrimidine biosynthesis. Targeting this metabolic pathway may therefore represent a potential mechanism underlying the cytotoxic effects of the extracts.

### 2.12. Gas Chromatography–Mass Spectrometry (GC–MS) Analysis

**Figure 9:**
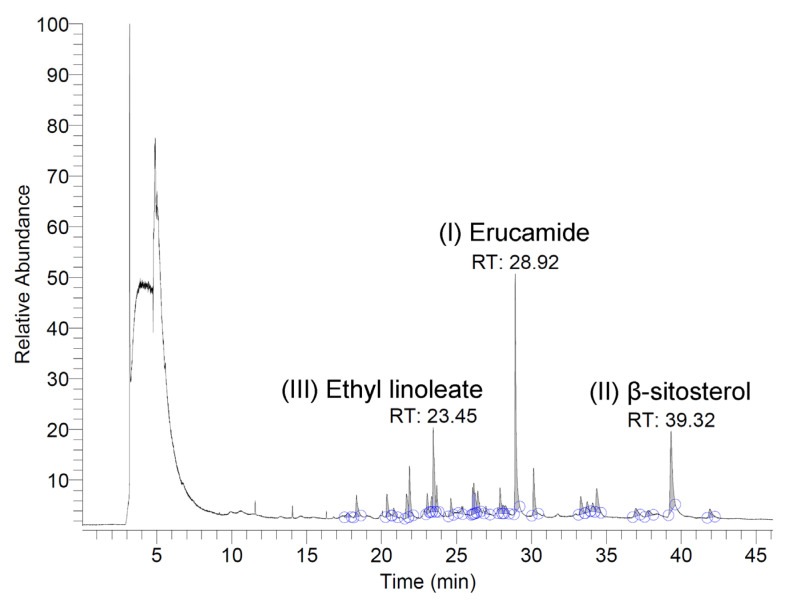
GC chromatogram. The extract was analyzed using GC–MS, and compounds were tentatively identified by matching the acquired spectra with mass spectral libraries. The three most abundant compounds detected were erucamide, β-sitosterol, and ethyl linoleate, with retention times of 28.92, 39.32, and 23.45 min, respectively.

The volatile and semi-volatile constituents of the *N. miranda* flower ethanol extract were analyzed using a GC–MS system. Compounds were tentatively identified by comparing the acquired mass spectra with those in the NIST and Wiley libraries, accepting only matches with a similarity index (SI) >900. Only volatile/semi-volatile components detectable under these conditions are reported here, as the analysis was intended as a preliminary chemical profile of *N. miranda* flowers—an underexplored plant tissue in this genus. The three most abundant detected compounds were erucamide, β-sitosterol, and ethyl linoleate, with retention times of 28.92, 39.32, and 23.45 min, respectively (Figure 9). The top nine compounds, each constituting more than 1.0% of the extract, were identified as follows: erucamide (19.36%), β-sitosterol (12.43%), ethyl linoleate (9.16%), 1-octacosanol (5.15%), ethyl palmitate (5.03%), palmitic acid (3.23%), neophytadiene (3.03%), phytol (2.07%), and linoleic acid (1.25%). It should be noted that erucamide is a known contaminant associated with plastic laboratory materials, and its occurrence here may not reflect a genuine plant metabolite. Furthermore, because GC–MS without derivatization primarily detects volatile and semi-volatile compounds, non-volatile metabolites such as polyphenols, amino acids, and triterpenoids were not captured in this analysis. These classes of compounds are likely to contribute substantially to the observed bioactivities and will be targeted in future studies using derivatization-based GC–MS and/or LC–MS approaches.

## 3. Discussion

The genus *Nepenthes*, known for its carnivorous traits, has traditionally been used in ethnomedicine for treating ailments such as stomach discomfort and fever [3]. Since Charles Darwin’s pioneering work on carnivorous plants, these species have attracted considerable attention for their evolutionary innovations, particularly their passive, pitcher-shaped traps that represent highly specialized morphological adaptations for nutrient acquisition [1,2,87]. In recent years, there has been growing scientific interest in exploring the pharmacological properties of *Nepenthes* species, especially through extracts obtained from pitchers, stems, and leaves, which have demonstrated promising bioactivities, including anticancer potential [6,7,8,9,10]. In contrast, the pharmacological effects of *Nepenthes* flowers remain largely unexplored. To address this gap, the present study utilized *N. miranda* as a model to evaluate the bioactive potential of its flower extract (Figure 1), with a specific focus on biomedical and cosmeceutical applications. To assess its suitability for therapeutic development, the TPC (Table 1) and TFC (Table 2) of the flower extracts were evaluated. Among the solvents tested (water, methanol, ethanol, and acetone), the ethanol extract yielded the highest levels of both TPC and TFC, indicating a rich presence of bioactive phytochemicals. These findings suggest that ethanol is the most effective and appropriate solvent for extracting pharmacologically active compounds from *N. miranda* flowers. Given its relatively low toxicity and general safety for biological applications, the ethanol-extracted flower fraction may serve as a promising candidate source for further investigation in natural-product-based drug discovery and development.

To the best of our knowledge, this study provides the first comprehensive characterization of the pharmacological properties of *N. miranda* flower extracts, including their antioxidant (Table 3), antibacterial (Table 4), anti-aging (Table 5 and Figure 2, Figure 3 and Figure 4), antidiabetic (Figure 5), and anticancer potential (Figure 6 and Figure 7). The ethanol extract of the flower exhibited notably high levels of TPC and TFC, which strongly correlated with its potent antioxidant capacity, as demonstrated by the DPPH radical scavenging assay. Since oxidative stress is a major contributor to the pathogenesis of chronic diseases [88,89,90] and skin aging [91,92,93,94], the observed antioxidant activity supports the potential application of this extract in both therapeutic and cosmetic settings. Beyond its antioxidative effects, the ethanol flower extract also exhibited broad-spectrum antibacterial activity, with inhibition zones reaching up to 29 mm against both Gram-positive (*S. aureus*) and Gram-negative (*E. coli*) bacteria. Most flavonoids exert antibacterial and astringent activities that help in infection control [95,96]. Given that flavonoids are known to exert antimicrobial effects by disrupting the cytoplasmic membrane, interfering with energy metabolism, and inhibiting nucleic acid synthesis [97], the high TFC in the ethanol extract likely contributes to its antibacterial efficacy. These findings suggest the presence of antimicrobial phytochemicals in the ethanol extract capable of suppressing bacterial growth, further supporting its potential use in preventing or managing skin infections and inflammation-related dermatological conditions.

Skin aging is a natural and progressive process [40,98] influenced by intrinsic and extrinsic factors, including lifestyle and environmental stressors, which can accelerate its onset and lead to premature dermal deterioration [27,28,29,32]. In this study, the ethanol extract of *N. miranda* flowers demonstrated promising potential in mitigating skin aging effects (Table 5). Enzyme inhibition assays relevant to cosmetic applications revealed that the extract significantly inhibited tyrosinase (IC_50_ = 48.58 μg/mL), elastase (IC_50_ = 1.77 μg/mL), and hyaluronidase (IC_50_ = 7.33 μg/mL), supporting its prospective use in maintaining skin elasticity, reducing hyperpigmentation, and enhancing moisture retention. The methanol extract also exhibited strong tyrosinase inhibitory activity, with an IC_50_ of 37.04 μg/mL (Figure 2). Based on the IC_50_ values, the anti-elastase (Figure 3) and anti-hyaluronidase (Figure 4) activities of the ethanol extract were especially potent. The IC_50_ values achieved surpassed those of the established positive controls EGCG (IC_50_ = 4.25 μg/mL) and myricetin (IC_50_ = 9.74 μg/mL), suggesting that the extract contains highly active constituents capable of strong enzyme inhibition. Accordingly, specific bioactive compound(s) within the ethanol extract, acting either individually or synergistically, may be responsible for these effects. Ongoing investigations in our laboratory aim to isolate and identify these active molecules to support their development into anti-aging skincare agents, potentially as ingredients in creams, serums, or adjunctive formulations. Furthermore, with nearly 120 species classified under the *Nepenthes* genus [3], it would be valuable to determine whether similar anti-aging activities are conserved across related species, offering broader potential for natural-product-based dermatological innovations.

Type 2 diabetes mellitus (T2DM), the most prevalent form of diabetes accounting for 90–95% of global cases, is a chronic metabolic disorder marked by insulin resistance and elevated blood glucose levels [23,24,25]. If poorly managed, it can lead to serious complications including hypertension, retinopathy, nephropathy, cardiovascular disease, and limb amputation [25]. One therapeutic approach to controlling postprandial blood glucose spikes involves inhibiting α-glucosidase, a digestive enzyme responsible for carbohydrate breakdown [99]. In this study, the ethanol extract of *N. miranda* flowers exhibited potent α-glucosidase inhibitory activity, with an IC_50_ of 24.53 μg/mL (Figure 5), which was more effective than the reference compounds acarbose (IC_50_ = 143.71 μg/mL) and quercetin (IC_50_ = 56.85 μg/mL). These findings suggest that the ethanol extract holds promise as a natural source for developing functional food supplements or antidiabetic agents aimed at managing T2DM.

Cancer remains one of the foremost causes of mortality worldwide, necessitating the continuous development of effective therapeutic strategies [49,50,51]. In addition to exhibiting significant biochemical activities, including the inhibition of tyrosinase, elastase, hyaluronidase, and α-glucosidase (Table 5), the flower extracts of *N. miranda* also demonstrated potent cytotoxic effects against A431 human epidermoid carcinoma cells, a model commonly used in skin cancer research. Among the tested extracts, the ethanol fraction stood out for its pronounced anticancer efficacy. It induced apoptosis in a dose-dependent manner, significantly suppressed cell migration, and markedly inhibited clonal proliferation, as evidenced by the clonogenic assay (Figure 6). Further mechanistic insights were gained through the comet assay (Figure 7), which revealed extensive DNA fragmentation in cells treated with the ethanol extract. The observed genotoxicity strongly suggests that the extract exerts its cytotoxic effects by inducing DNA damage, thereby triggering apoptosis. This mechanism, which involves inducing DNA damage and subsequently triggering apoptosis, is commonly observed and aligns with the action of several plant-derived anticancer agents [100,101,102,103]. Accordingly, these findings underscore the potential of *N. miranda* flower ethanol extract as a candidate for topical or systemic therapies targeting skin cancers such as squamous cell carcinoma. Further studies may therefore focus directly on confirming the molecular targets and pathways involved.

This study provides the first evidence that *N. miranda* flower extracts inhibit α-glucosidase (IC_50_ = 24.53 μg/mL), with potency exceeding that of acarbose and quercetin. The observed elastase, tyrosinase, and hyaluronidase inhibition (Table 5) was comparable to that previously reported for *N. miranda* stem extracts [6]. Notably, using floral tissues offers a sustainable alternative to stem harvesting, enabling continued exploration of *Nepenthes* bioactives without harming the plant.

Our previous works have determined the crystal structures of dihydroorotase in complex with various non-substrate ligands and inhibitors, including malate [104], 5-fluoroorotate [105], plumbagin [10], 5-aminouracil [106], and 5-fluorouracil [86]. In this study, we discovered that the flower extracts of *N. miranda* exhibit notable inhibitory effects on the enzymatic activity of human dihydroorotase, providing a potential mechanistic basis for their observed anticancer properties. Among the tested extracts, the ethanol and methanol fractions demonstrated significant inhibition, with IC_50_ values of 25.11 μg/mL and 36.48 μg/mL, respectively (Figure 8). Dihydroorotase plays a critical role as the third enzyme in the de novo pyrimidine biosynthesis pathway, which is essential for nucleotide production in rapidly proliferating cells. Given that cancer cells exhibit increased nucleotide demand to sustain their accelerated growth, disruption of this pathway can severely impair their proliferation. Consequently, dihydroorotase has emerged as a promising target for the development of anticancer, antimalarial, and antimicrobial agents. The inhibition of dihydroorotase by the *N. miranda* flower extracts may therefore represent a plausible biochemical mechanism contributing to their cytotoxicity against A431 epidermoid carcinoma cells (Figure 6). Similar inhibitory activity has been reported for the acetone extract of the roots of *Sarracenia purpurea*, another carnivorous plant species, with an IC_50_ exceeding 30 μg/mL. However, the ethanol flower extract of *N. miranda* exhibited even greater potency, suggesting its potential as a source of alternative or complementary therapeutics for skin cancers, particularly squamous cell carcinoma. Although *S. purpurea* and *N. miranda* differ in many botanical and ecological aspects, these findings support the continued exploration of carnivorous plant extracts as promising sources of novel bioactive compounds targeting key metabolic pathways in cancer cells.

In the present study, the ethanol extract from *N. miranda* flowers exhibited notable in vitro cytotoxicity and apoptosis-inducing effects in skin cancer cells. These findings suggest that certain constituents of the extract may have the potential to target cancer cell viability. However, as normal skin control cells were not tested, we cannot draw conclusions on cancer-specific selectivity. Further studies, including assays in non-cancerous cell models, are necessary to determine the extract’s therapeutic potential and safety profile.

In this study, GC–MS was applied primarily to obtain a preliminary volatile and semi-volatile profile of the *N. miranda* flower ethanol extract, as floral tissues in *Nepenthes* species remain chemically underexplored. Although the method is well suited for detecting compounds such as fatty acid esters, sterols, and other volatile/semi-volatile metabolites, it does not capture the majority of non-volatile constituents (e.g., phenolic acids, flavonoids, triterpenoids, and amino acids) that are likely to underlie much of the observed antioxidant, antimicrobial, anti-aging, and cytotoxic activities. The detection of erucamide, a compound widely recognized as a common contaminant from plastic labware, is noted here as a probable artifact rather than a genuine plant metabolite. Future work will focus on targeted profiling of these non-volatile bioactive constituents using derivatization-based GC–MS and LC–MS approaches. Such complementary analyses will help establish direct correlations between specific compound classes and the biological activities reported here, thereby providing a more comprehensive chemical characterization of *N. miranda* flowers.

In conclusion, the flower extracts of *N. miranda*, particularly the ethanol fraction, represent a promising source of multifunctional bioactive compounds. The demonstrated antioxidant, antimicrobial, anti-aging, antidiabetic, and anticancer potential suggests that these extracts could serve as alternative or complementary therapeutics for managing skin disorders and regulating carbohydrate metabolism. The GC–MS analysis identified several bioactive constituents such as β-sitosterol, ethyl linoleate, 1-octacosanol, ethyl palmitate, palmitic acid, neophytadiene, phytol, and linoleic acid. These findings not only broaden our understanding of carnivorous plants as reservoirs of novel therapeutic agents but also emphasize the underexplored potential of floral tissues in phytochemical and pharmacological research.

## 4. Materials and Methods

### 4.1. Materials

All solvents and chemicals were obtained from Sigma-Aldrich (St. Louis, MO, USA). The *Escherichia coli* strain BL21(DE3) pLysS (Novagen, Worcestershire, UK) was used for recombinant protein expression. The A431 cell line was kindly provided by Dr. Kuo-Ting Chang (Taoyuan General Hospital, Taiwan). A431 cells were grown (37 °C, 5% CO_2_) in a DME medium (GibcoTM; Thermo Fisher Scientific, Waltham, MA, USA) with 10% FBS, 100 units/mL penicillin, and 100 μg/mL streptomycin.

### 4.2. Expression and Purification of the Recombinant Protein

The plasmid for human dihydroorotase expression was constructed as previously described [107]. Recombinant human dihydroorotase was purified using Ni-NTA affinity chromatography [80]. Briefly, *E. coli* BL21(DE3) cells transformed with the plasmid were induced with 1 mM isopropyl β-D-1-thiogalactopyranoside (IPTG) for protein overexpression. The His-tagged protein was purified using Ni^2+^-affinity chromatography (HiTrap HP; GE Healthcare Bio-Sciences, Chicago, IL, USA) and eluted with elution buffer (20 mM Tris–HCl, 250 mM imidazole, and 0.5 M NaCl, pH 7.9). The eluted protein was dialyzed against storage buffer (40 mM Tris-HCl and 50 mM NaCl, pH 7.5) for downstream applications. Protein purity was confirmed to be >97% by SDS–PAGE (Mini-PROTEAN Tetra System; Bio-Rad, Hercules, CA, USA).

### 4.3. Plant Materials and Extract Preparations

Extracts of the flower, pitcher, stem, and leaf of *N. miranda* were prepared using 100% methanol, ethanol, acetone, and water. This plant was obtained from the Guoguang Flower Market and the Taiwan Provincial Flower Marketing Cooperative and verified by Dr. Zhong-Bao Zhang in June 2023. The collected plant parts were air-dried, cut into smaller fragments, and ground into a fine powder. For extraction, 1 g of powdered material was combined with 100 mL of the solvent in a 250 mL Erlenmeyer flask and agitated on an orbital shaker for 5 h. The mixture was filtered through a 0.45 μm membrane, and the solvent was evaporated using a hot air circulation oven set at 40 °C. The dried extracts were stored at –80 °C until use. The yields of the obtained extracts are presented in Table 6. For experimental uses, extract powders were dissolved in 20% DMSO to create a 20 mg/mL stock solution. This stock was further diluted in culture medium for cytotoxicity testing or in assay buffer for enzyme analyses. A final concentration of 0.2% DMSO was used as the negative control.

### 4.4. Total Phenolic Content and Flavonoid Content

Total phenolic content (TPC) was quantified using the Folin–Ciocalteu reagent according to a previously described protocol [84]. The intensity of the resulting blue coloration was measured at 750 nm using a UV/VIS spectrophotometer (Hitachi U-3300, Hitachi High-Technologies, Tokyo, Japan). Results were calculated based on a gallic acid standard curve and expressed as milligrams of gallic acid equivalents per gram of dry plant material. For total flavonoid content (TFC), the aluminum chloride colorimetric assay was employed following a reported method [85]. Absorbance readings for both the plant extracts and quercetin standards were recorded at 510 nm using the same spectrophotometer. TFC values were expressed as milligrams of quercetin equivalents per gram of dry weight. Standard curves for gallic acid and quercetin were prepared within a concentration range of 0–500 μg/mL. All measurements were performed in triplicate, and data are presented as mean ± standard deviation.

### 4.5. Antioxidant Activity Analysis

The antioxidant potential of the plant extracts was assessed using the 1,1-diphenyl-2-picrylhydrazyl (DPPH) radical scavenging assay, following a previously established method [108]. Absorbance readings were taken at 517 nm to evaluate the reduction in DPPH radical concentration upon reaction with the extracts. The scavenging activity (%) was calculated using the following formula: % Inhibition = [(Absorbance of control − Absorbance of sample)/Absorbance of control] × 100. All results are presented as the mean ± standard deviation of at least three independent assays. L-ascorbic acid, used as the positive control, exhibited an IC_50_ value of 28.6 ± 0.2 μg/mL.

### 4.6. Agar Well Diffusion Assay

The antibacterial properties of the plant extracts were evaluated using the agar well diffusion method [109]. Bacterial suspensions of *E. coli* and *S. aureus* were adjusted to a 0.1 McFarland standard and evenly spread onto sterile Petri dishes containing 60 mL of Mueller–Hinton agar (Sigma-Aldrich, St. Louis, MO, USA). Plant extracts were prepared in 20% DMSO, and 10 mg (100 μL of 100 mg/mL) of each extract was applied into wells created in the agar. The plates were then incubated at 37 °C for 12 h. Antibacterial activity was determined by measuring the diameter of the inhibition zones surrounding each well, with larger zones indicating greater efficacy. Ampicillin (100 μg; 100 μL of 1 mg/mL) and 20% DMSO were used as positive and negative controls, respectively. No inhibition zone was produced by 20% DMSO. Results are expressed as the mean ± standard deviation from at least three independent experiments.

### 4.7. Human Dihydroorotase Inhibition

Dihydroorotase activity was assessed using a spectrophotometric method that monitors substrate hydrolysis in real time [110]. The enzymatic reaction was initiated by adding 15 μg of purified human dihydroorotase to a 2 mL reaction mixture containing 0.25 mM dihydroorotate and the plant extract in 100 mM Tris–HCl buffer (pH 8.0) at 25 °C. Enzyme activity was monitored by measuring the decline in absorbance at 230 nm using a UV/Vis spectrophotometer (Hitachi U-3300, Tokyo, Japan).

### 4.8. Elastase Inhibition

Elastase inhibition was evaluated using a modified spectrophotometric assay [111]. The reaction was performed in 200 mM Tris buffer (pH 8.0) with porcine pancreatic elastase (2 μg/mL) and 0.8 mM N-Succinyl-Ala-Ala-Ala-p-nitroanilide (AAAPVN) as the substrate. Test extracts (0–100 μg/mL) were preincubated with the enzyme for 15 min before substrate addition. EGCG served as the positive control, while 10% DMSO was used as the negative control. Absorbance at 405 nm was recorded over 20 min in 96-well plates. Inhibition was calculated as follows: % inhibition = [(A_control − A_sample)/A_control] × 100.

### 4.9. Tyrosinase Inhibition

Tyrosinase inhibition was measured using a modified dopachrome assay with L-DOPA as the substrate [112]. Plant extracts, prepared in 20% DMSO and diluted in 0.1 M phosphate buffer (pH 6.8), were mixed with mushroom tyrosinase (200 U/mL) and 2.5 mM L-DOPA in 96-well plates. After 30 min incubation at 37 °C, absorbance was recorded at 475 nm. Blanks without L-DOPA were used for background correction. Quercetin served as the positive control, and 10% DMSO was used as the negative control. Inhibition (%) was calculated as: [(A_control − A_sample)/A_control] × 100.

### 4.10. Hyaluronidase Inhibition

Hyaluronidase inhibition was evaluated using a modified spectrophotometric assay [113]. Briefly, 25 μL of extract (0–100 μg/mL) was pre-incubated with bovine testis hyaluronidase (0.4 U/mL; H3506, Sigma-Aldrich, St. Louis, MO, USA) in phosphate buffer (pH 7.0) containing NaCl and BSA for 10 min at 37 °C. After sequential additions of acidic phosphate buffer and 0.03% hyaluronic acid, the mixture was incubated for 45 min. The reaction was stopped with an acidic albumin solution, and absorbance was measured at 600 nm. Myricetin was used as the positive control; 10% DMSO served as the negative control. Inhibition (%) was calculated as follows: [(A_control − A_sample)/A_control] × 100.

### 4.11. α-Glucosidase Inhibition

α-Glucosidase inhibition was assessed by pre-incubating 25 μL of extract with 100 μL of α-glucosidase (0.2 U/μL) in 0.1 M phosphate buffer (pH 6.8) at 37 °C for 10 min. The reaction was initiated by adding 25 μL of 5 mM p-nitrophenyl-α-D-glucopyranoside and continued for 20 min at 37 °C. Absorbance was measured at 410 nm. Controls used buffer in place of extract.

### 4.12. MTT Cell Viability Assay

Cell viability was assessed via the MTT assay [114]. Cells were seeded in 96-well plates (5 × 10^3^ cells/well) and treated with serial dilutions of the extract. After 24 h, 30 μL of MTT solution (5 mg/mL in PBS) was added and incubated for 4 h at 37 °C. Formazan crystals were solubilized with 100 μL of DMSO, and absorbance was measured at 540 nm. Experiments were performed in triplicate.

### 4.13. Chromatin Condensation Assay

To assess apoptosis, cells were seeded in 96-well plates (5 × 10^3^ cells/well) and allowed to attach for 24 h. Following treatment with the plant extract for 24 h, the cells were rinsed with PBS and stained with Hoechst 33342 (1 μg/mL) [115] for 10 min in the dark. Fluorescent images were captured using the ImageXpress Pico system (Molecular Devices, San Jose, CA, USA) with DAPI filters, and analysis was conducted using CellReporterXpress Version 2 software.

### 4.14. Clonogenic Formation Assay

To examine the extract’s effect on long-term cell proliferation, a colony formation assay was performed [116]. Cells were plated in 6-well plates at a density of 1.5 × 10^3^ cells per well and allowed to adhere overnight. After treatment with the plant extract for 7 to 8 days, the cells were washed with PBS, fixed using methanol, and stained with 0.5% crystal violet for 20 min. Colony numbers were then quantified under a light microscope to evaluate the extent of growth inhibition.

### 4.15. Wound-Healing Assay

Cell migration was assessed using the wound-healing assay [117], which monitors two-dimensional collective movement. A scratch was made across a confluent monolayer of A431 cells cultured in serum-reduced medium for 6 h. Following scratch creation with a pipette tip and PBS washing, cells were treated with the plant extract for 24 h, and gap closure was evaluated to determine migration inhibition.

### 4.16. Comet Assay

The comet assay was performed with slight modifications from established protocols [118]. Cells were mixed with 1% low-melting-point agarose (1:10) and layered onto pre-coated slides. Slides were immersed in lysis buffer (pH 10) for 90 min in the dark. DNA unwinding and electrophoresis were carried out in alkaline buffer (pH 13) at 1.25 V/cm for 25 min. Slides were rinsed, fixed in 95% ethanol, air-dried, and stained with propidium iodide (2.5 μg/mL in PBS). Fluorescent images were captured using a ZEISS Axio Imager A2, and at least 100 cells per slide were analyzed using Comet Score™ to determine tail DNA percentage and tail moment.

### 4.17. Gas Chromatography–Mass Spectrometry

The chemical composition of the *N. miranda* flower ethanol extract was analyzed using gas chromatography–mass spectrometry (GC–MS). The analysis was performed on a system equipped with an Rxi-5MS fused silica capillary column (30 m × 0.25 mm I.D., 0.25 μm film thickness). The carrier gas was helium, maintained at a constant flow rate of 1.0 mL/min. A split injection mode was employed with a split ratio of 5:1. One microliter of the ethanol extract (70 μL stock) was injected into the GC-MS system. The inlet temperature was maintained at 300 °C. The GC oven temperature program was as follows: initial temperature set at 40 °C, followed by an increase at a rate of 10 °C/min to 300 °C, with a final hold at 300 °C for 20 min. No solvent delay was applied. The mass spectrometer was operated in electron ionization (EI) mode over a mass range of 29–650 amu. The obtained chromatographic peaks were identified by comparing their mass spectra with those in the NIST MS library. A solvent blank (ethanol only) was used for background subtraction and to ensure specificity of detected compounds. Only compounds in the extract with a similarity index (SI) greater than 900, as identified by GC-MS, are reported here.

### 4.18. Statistical Analysis

Experiments were conducted in triplicate, and results are presented as mean ± standard deviation (SD). Statistical analysis was performed using GraphPad Prism 8.3.0 (GraphPad Software Inc., San Diego, CA, USA). One-way ANOVA followed by post hoc tests was used to assess statistical differences among groups, with *p*-values less than 0.05 being considered significant.

## Figures and Tables

**Table 1:** TPC of *N. miranda* extracts.

Solvent	Flower	Leaf	Stem	Pitcher
Water	8.6 ± 0.4 ^e^	5.2 ± 0.6 ^f^	5.8 ± 0.2 ^f^	3.7 ± 0.5 ^f^
Methanol	17.9 ± 0.7 ^a^	12.8 ± 0.6 ^c^	14.6 ± 0.6 ^c^	12.5 ± 0.4 ^c^
Ethanol	18.2 ± 0.6 ^b^	12.5 ± 0.5 ^c^	13.6 ± 0.1 ^c^	9.6 ± 0.3 ^e^
Acetone	9.5 ± 0.4 ^e^	10.9 ± 0.2 ^d^	16.2 ± 0.7 ^b^	11.3 ± 0.5 ^d^

TPC was quantified using the modified Folin–Ciocalteu method. The absorbance of blue color developed was measured at 750 nm by using a UV/VIS spectrophotometer. The results were compared with the standard curves of gallic acid (GAE) and were expressed as mg equivalent/g dry weight (mg GAE/g). Different letters (a–f) indicate statistically significant differences (*p* < 0.05) according to Tukey’s multiple comparisons test.

**Table 2:** TFC of *N. miranda* extracts.

Solvent	Flower	Leaf	Stem	Pitcher
Water	10.2 ± 0.5 ^i^	7.3 ± 0.5 ^i^	24.7 ± 0.8 ^h^	2.9 ± 0.4 ^j^
Methanol	53.9 ± 0.7 ^c^	30.4 ± 0.8 ^g^	63.4 ± 1.0 ^b^	27.9 ± 1.2 ^g^
Ethanol	68.9 ± 1.1 ^a^	44.6 ± 1.3 ^d^	67.8 ± 1.1 ^a^	33.6 ± 1.1 ^f^
Acetone	41.6 ± 1.5 ^e^	61.2 ± 1.1 ^b^	62.4 ± 0.4 ^b^	42.8 ± 1.5 ^e^

TFC was quantified using the aluminum chloride calorimetric method. The absorbance of extracts and standard solutions was measured at 510 nm. The results were expressed as mg of QUE equivalent/g dry weight (mg QUE/g). Different letters (a–j) indicate statistically significant differences (*p* < 0.05) according to Tukey’s multiple comparisons test.

**Table 3:** Antioxidant activity of the flower extracts.

	IC_50_ (μg/mL)		
	Methanol	Ethanol	Acetone
Flower	70.7 ± 1.0 ^b^	66.9 ± 1.2 ^a^	92.0 ± 2.4 ^c^

IC_50_ values were calculated from the titration curves of the DPPH assay by determining the concentration of extract required to achieve 50% radical scavenging activity. L-Ascorbic acid as a positive control had an IC_50_ value of 28.6 ± 0.2 μg/mL. The IC_50_ value for the water extract could not be determined due to its low antioxidant activity. Different letters (a–c) indicate statistically significant differences (*p* < 0.05) according to Tukey’s multiple comparisons test.

**Table 4:** Antibacterial activities of the flower extracts.

	Water	Methanol	Ethanol	Acetone
*E. coli*	0 ± 0 ^d^	16 ± 1 ^b^	29 ± 2 ^a^	8 ± 1 ^c^
*S. aureus*	0 ± 0 ^d^	13 ± 1 ^b^	27 ± 1 ^a^	7 ± 1 ^c^

Ampicillin (100 μg; 100 μL of 1 mg/mL) and 20% DMSO served as the positive and negative controls, respectively. The inhibition zones produced by ampicillin were 14 ± 1 mm for *E. coli* and 26 ± 2 mm for *S. aureus*. No inhibition zone was produced by 20% DMSO. Different letters (a–d) indicate statistically significant differences (*p* < 0.05) according to Tukey’s multiple comparisons test.

**Table 5:** Inhibition effects of the flower extracts.

	IC_50_ (μg/mL)			
Inhibitor	Tyrosinase	Elastase	Hyaluronidase	α-Glucosidase
Water extract	ND	20.59 ± 1.22 ^c^	49.28 ± 2.82 ^c^	96.69 ± 2.22 ^d^
Methanol extract	37.04 ± 2.33 ^a^	17.06 ± 0.80 ^b^	8.58 ± 0.62 ^a^	67.18 ± 1.73 ^c^
Ethanol extract	48.58 ± 1.37 ^b^	1.77 ± 0.19 ^a^	7.33 ± 0.15 ^a^	24.53 ± 0.75 ^a^
Acetone extract	172.43 ± 2.24 ^c^	77.94 ± 1.68 ^d^	41.97 ± 2.18 ^b^	ND
Quercetin	39.07 ± 1.92 ^a^			56.85 ± 1.48 ^b^
EGCG		4.25 ± 0.13 ^a^		
Myricetin			9.74 ± 0.48 ^a^	
Acarbose				143.71 ± 2.22 ^e^

ND, not determined. Different superscript letters within each column indicate statistically significant differences between groups (*p* < 0.05, Tukey’s test).

**Table 6:** The yields of the extracts.

Solvent	Flowers	Leaves	Stems	Pitchers
Water	15.84 ± 1.1 ^a^	31.47 ± 1.1 ^c^	30.75 ± 1.3 ^c^	11.61 ± 0.8 ^a^
Methanol	12.41 ± 0.8 ^a^	24.28 ± 0.5 ^b^	25.62 ± 0.7 ^b^	11.89 ± 0.8 ^a^
Ethanol	11.54 ± 0.8 ^a^	22.11 ± 0.6 ^b^	23.57 ± 0.9 ^b^	10.76 ± 0.9 ^a^
Acetone	7.70 ± 0.3 ^a^	11.15 ± 0.5 ^a^	13.16 ± 0.6 ^a^	9.35 ± 0.5 ^a^

Data are expressed as mean ± SD (*n* = 3). Different superscript letters within each column indicate statistically significant differences between groups (*p* < 0.05, Tukey’s test).

## Data Availability

Data are contained within the article.
